# Supplementary material for: Arsenic is a potent co-mutagen of ultraviolet light
Source: Commun Biol. 2023 Dec 16;6:1273. doi: 10.1038/s42003-023-05659-4 (PMC10725444; doi:10.1038/s42003-023-05659-4)
Supplement: Supplementary file 2 — Description of Additional Supplementary Files [file 42003_2023_5659_MOESM2_ESM.pdf]

Description of Additional Supplementary Files

File name: Supplementary Data 1
Description: Source data for all figures and supplementary figures.
