# Supplementary material for: Arsenic is a potent co-mutagen of ultraviolet light
Source: Commun Biol. 2023 Dec 16;6:1273. doi: 10.1038/s42003-023-05659-4 (PMC10725444; doi:10.1038/s42003-023-05659-4)
Supplement: Supplementary file 4 — Reporting Summary [file 42003_2023_5659_MOESM4_ESM.pdf]

nature portfolio

Corresponding author(s): Ludmil B. Alexandrov; Ke Jian Liu

Last updated by author(s): Nov 20, 2023

Reporting Summary

Nature Portfolio wishes to improve the reproducibility of the work that we publish. This form provides structure for consistency and transparency in reporting. For further information on Nature Portfolio policies, see our Editorial Policies and the Editorial Policy Checklist.

Statistics

For all statistical analyses, confirm that the following items are present in the figure legend, table legend, main text, or Methods section.

n/a	Confirmed	
☐	☒	The exact sample size (*n*) for each experimental group/condition, given as a discrete number and unit of measurement
☐	☒	A statement on whether measurements were taken from distinct samples or whether the same sample was measured repeatedly
☐	☒	The statistical test(s) used AND whether they are one- or two-sided<br>*Only common tests should be described solely by name; describe more complex techniques in the Methods section.*
☐	☒	A description of all covariates tested
☐	☒	A description of any assumptions or corrections, such as tests of normality and adjustment for multiple comparisons
☐	☒	A full description of the statistical parameters including central tendency (e.g. means) or other basic estimates (e.g. regression coefficient) AND variation (e.g. standard deviation) or associated estimates of uncertainty (e.g. confidence intervals)
☐	☒	For null hypothesis testing, the test statistic (e.g. *F*, *t*, *r*) with confidence intervals, effect sizes, degrees of freedom and *P* value noted<br>*Give P values as exact values whenever suitable.*
☒	☐	For Bayesian analysis, information on the choice of priors and Markov chain Monte Carlo settings
☒	☐	For hierarchical and complex designs, identification of the appropriate level for tests and full reporting of outcomes
☒	☐	Estimates of effect sizes (e.g. Cohen's *d*, Pearson's *r*), indicating how they were calculated

Our web collection on statistics for biologists contains articles on many of the points above.

Software and code

Policy information about availability of computer code

Data collection	In addition to the generate genomics data, data were collected from Pancancer Analysis of Whole Genomes (PCAWG) of the International Cancer Genome Consortium (ICGC) based on their official data release.<br><br>Somatic mutations in whole-genome sequencing data were identified using our ensemble variant calling pipeline, which is freely available under the permissive 2-clause BSD license at: https://github.com/AlexandrovLab/EnsembleVariantCallingPipeline. All other computational tools utilized in this publication have been previously published and can be access through their respective publications:<br>BWA (v.0.7.17), GATK4 (v.4.1.4.1-0), MuSE (v.1.0), Strelka (v.2.9.10), VarScan (v2.4.3), VEP (Ensembl API 96).
Data analysis	R (v.3.6.0 to v.4.2.0), samtools (v.1.9), BWA (v.0.7.17), VEP (Ensembl API 96), IGV (v.2.6.2).<br><br>R packages: ggplot2 (v.3.3.6), easyGgplot2 (v.1.0.0.9000), ComplexHeatmap (v.2.12.0), circlize (v.0.4.15), ggpubr (v.0.4.0), corrr (v.0.4.3), lsa (v.0.73.3). In addition, RColorBrewer, brew, reshape2, dplyr, plyr, GenomicRanges, stringr, tidyr, tidyverse, hrbrthemes, data.table, readxl.

For manuscripts utilizing custom algorithms or software that are central to the research but not yet described in published literature, software must be made available to editors and reviewers. We strongly encourage code deposition in a community repository (e.g. GitHub). See the Nature Portfolio guidelines for submitting code & software for further information.

Data

Policy information about availability of data

All manuscripts must include a data availability statement. This statement should provide the following information, where applicable:

- Accession codes, unique identifiers, or web links for publicly available datasets
- A description of any restrictions on data availability
- For clinical datasets or third party data, please ensure that the statement adheres to our policy

All whole-genome and duplex sequencing data have been deposited to Sequence Read Archive (SRA) and can be downloaded using accession numbers: PRJNA909329 (human samples) and PRJNA910941 (mouse samples). For mouse samples, we used the GRCm38 reference genome. For human samples, we used the GRCh38 reference genome. We employed the dbSNP142 for germline mutations detection in mouse samples, and dbSNP155 for human samples.

Human research participants

Policy information about studies involving human research participants and Sex and Gender in Research.

Reporting on sex and gender	N/A
Population characteristics	N/A
Recruitment	N/A
Ethics oversight	N/A

Note that full information on the approval of the study protocol must also be provided in the manuscript.

Field-specific reporting

Please select the one below that is the best fit for your research. If you are not sure, read the appropriate sections before making your selection.

☒ Life sciences ☐ Behavioural & social sciences ☐ Ecological, evolutionary & environmental sciences

For a reference copy of the document with all sections, see nature.com/documents/nr-reporting-summary-flat.pdf

Life sciences study design

All studies must disclose on these points even when the disclosure is negative.

Sample size	No statistical methods were applied to predetermine sample size for cell or mouse experiments. For N/TERT1 experiments 2-3 biological replicates (individual clones) were performed. For SKH-1 mouse experiments 3-4 biological replicates (individual animals) were performed each.
Data exclusions	One UVR sample was excluded in the N/TERT1 experiment due to the observation that this sample was hypermutated by APOBEC activity, which can arise randomly and episodically in cells (PMID: 30849372), was detected and was likely responsible for the exacerbation of mutations in this clone.
Replication	Multiple clones (for N/TERT1 experiments) per condition and tumors from different animals (SKH-1 mouse experiments) were included to ensure reproducibility. For cytotoxicity experiments two biological replicates with four technical replicated per exposure condition were included for reproducibility.
Randomization	For N/TERT1 experiments clones were randomly selected for expansion and sequencing. For SKH-1 mouse experiments the mice were randomly placed in the treatment groups and random tumors from each mouse group were selected for sequencing.
Blinding	Investigators were blinded for the analysis of the cytotoxicity experiments. Investigators were not blinded for analysis of the mutation data because the results are quantitative and did not require subjective judgment or interpretation from the invvestigators.

Reporting for specific materials, systems and methods

We require information from authors about some types of materials, experimental systems and methods used in many studies. Here, indicate whether each material, system or method listed is relevant to your study. If you are not sure if a list item applies to your research, read the appropriate section before selecting a response.

Materials & experimental systems

n/a	Involved in the study
☒	☐ Antibodies
☐	☒ Eukaryotic cell lines
☒	☐ Palaeontology and archaeology
☐	☒ Animals and other organisms
☒	☐ Clinical data
☒	☐ Dual use research of concern

Methods

n/a	Involved in the study
☒	☐ ChIP-seq
☒	☐ Flow cytometry
☒	☐ MRI-based neuroimaging

Eukaryotic cell lines

Policy information about cell lines and Sex and Gender in Research

Cell line source(s)	N/TERT1 cells used in this study were obtained from Dickson, M. A. et al. Human keratinocytes that express hTERT and also bypass a p16INK4a-enforced mechanism that limits life span become immortal yet retain normal growth and differentiation characteristics. Molecular and cellular biology 20, 1436-1447 (2000).
Authentication	Cells were visually assessed for morphological changes at each feeding and passage. Upon thawing and after every 3 months of continuous culture a chromosome spread was analyzed for any gross chromosomal changes.
Mycoplasma contamination	Cells were routinely tested for mycoplasma using the MycoAlert mycoplasma testing kit from Lonza and no contmaination was detected.
Commonly misidentified lines (See ICLAC register)	No commonly misidentified cell lines were used in this study.

Animals and other research organisms

Policy information about studies involving animals; ARRIVE guidelines recommended for reporting animal research, and Sex and Gender in Research

Laboratory animals	SKH-1 hairless mouse; 21 – 25 days old; purchased from Charles River Laboratories
Wild animals	Does not apply
Reporting on sex	All female mice were used in this study and selected for ease of housing as this was a pilot project with the possibility of further investigation using both sexes.
Field-collected samples	Does not apply
Ethics oversight	Oversight by UNM IACUC protocol # 22-201244-HSC

Note that full information on the approval of the study protocol must also be provided in the manuscript.
